# A Rapid Method for Label-Free Enrichment of Rare Trophoblast Cells from Cervical Samples

**DOI:** 10.1038/s41598-019-48346-3

**Published:** 2019-08-20

**Authors:** Christina M. Bailey-Hytholt, Sumaiya Sayeed, Morey Kraus, Richard Joseph, Anita Shukla, Anubhav Tripathi

**Affiliations:** 1 Center for Biomedical Engineering, School of Engineering, Brown University, Providence, RI 02912 USA; 2 PerkinElmer, 940 Winter St, Waltham, Massachusetts, 02451 USA

**Keywords:** Diagnosis, Biomedical engineering, Translational research

## Abstract

Extravillous trophoblasts (EVTs) have the potential to provide the entire fetal genome for prenatal testing. Previous studies have demonstrated the presence of EVTs in the cervical canal and the ability to retrieve a small quantity of these cells by cervical sampling. However, these small quantities of trophoblasts are far outnumbered by the population of cervical cells in the sample, making isolation of the trophoblasts challenging. We have developed a method to enrich trophoblast cells from a cervical sample using differential settling of the cells in polystyrene wells. We tested the addition of small quantities of JEG-3 trophoblast cell line cells into clinical samples from standard Pap tests taken at 5 to 20 weeks of gestation to determine the optimal work flow. We observed that a 4 min incubation in the capture wells led to a maximum in JEG-3 cell settling on the surface (71 ± 10% of the initial amount added) with the removal of 91 ± 3% of the cervical cell population, leading to a 700% enrichment in JEG-3 cells. We hypothesized that settling of mucus in the cervical sample affects the separation. Finally, we performed a proof-of-concept study using our work flow and CyteFinder cell picking to verify enrichment and pick individual JEG-3 and trophoblast cells free of cervical cells. Ultimately, this work provides a rapid, facile, and cost-effective method for enriching native trophoblasts from cervical samples for use in subsequent non-invasive prenatal testing using methods including single cell picking.

## Introduction

Extravillous trophoblasts (EVTs) are cells that originate from the placenta and invade the endometrium. These rare cells have the potential to enhance non-invasive prenatal testing (NIPT)^1,2^. NIPT is an important method for detecting fetal complications as it has lower cost and risks compared to invasive measures^3^. Biomarker techniques and cell free fetal DNA from patient blood samples have shown promise in some prenatal testing, particularly for aneuploidy screening^4^. However, these techniques are limited by the genetic information available in the samples. The capture of the entire fetal genome contained in intact fetal cells would be a significant improvement over current testing capabilities.

During trophoblast invasion EVTs enter the endocervical canal^5^, which can be sampled by a cervical swab. Previous studies have demonstrated the ability for trophoblast retrieval and isolation from the cervix (TRIC)^5–10^. With the frequency of one EVT per 2,000 cervical cells^5^, novel isolation methods are needed for downstream testing to provide a quality sample that is not overwhelmed by maternal cells. Common cell separation techniques are based on cell density, size, shape, piezoelectric effects, electric capacitance, magnetic susceptibility, hydrodynamic properties, and affinity to antibodies^11,12^. However, many of these techniques are not suitable for capturing EVTs with minimal cell loss or equipped to handle the cervical matrix^13^. In our investigation, we have utilized the intrinsic properties of these rare cells and their matrix in order to yield an EVT enriched sample based on differential settling of the cells in polystyrene wells.

In order to isolate putative fetal cells, studies to date have commonly used antibodies for human leukocyte antigen G (HLA-G), cytokeratin, beta human chorionic gonadotrophin (β-hCG), and X and Y chromosome probing using fluorescence *in situ* hybridization (FISH) and polymerase chain reaction (PCR)^8,14,15^. Isolation methods in the literature have used HLA-G coupled to magnetic beads to elute trophoblast cells from the maternal cell population^9,10^. However, any amount of maternal cells or DNA present in a sample can pose further challenge during analysis of the genome. Single cell picking, in which a single fetal cell is identified and selected from a mixed population of both maternal and fetal cells, is one advantageous strategy to eliminate the presence of maternal cells and isolate pure trophoblasts^16,17^. This is a similar approach to previous investigations aiming to isolate rare tumor cells^18,19^. However, a major issue of picking a single trophoblast cell from a cervical sample with no clean-up is the overwhelming density of cervical cells, which makes picking challenging and near impossible. Our strategy allows enrichment to a degree that improves the ability to pick and isolate a single trophoblast cell while effectively removing maternal contamination.

**Figure 1:**
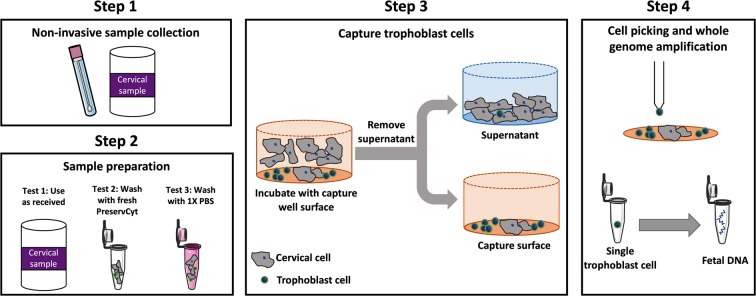
Workflow for trophoblast enrichment. Step 1 is the collection of trophoblast cells from the cervical canal using a cervical swab test method. Step 2 is the sample preparation by either using the sample as received from the clinic, washing with fresh PreservCyt^®^, or washing with 1× PBS. Step 3 is the enrichment of the cells using the workflow developed in this study. Step 4 is acquiring the fetal information by single cell picking and whole genome amplification.

The goal of this work was to enrich a cervical sample to increase the trophoblast frequency for optimal single cell picking. In this study we provide a facile workflow that eliminates at least 90% of squamous cervical cells and captures at least 70% of fetal cells (Fig. 1). We used cervical cells from clinical Papanicolaou (Pap) tests stored in ThinPrep^®^ PreservCyt^®^ and supplemented with a known number of JEG-3 cells (a common trophoblast cell line) for parameter optimization. To achieve enrichment, we allowed the JEG-3 and cervical cells to settle in a polystyrene well for a variable amount of time. After the settling time, we removed the supernatant, which contained a large majority of cervical cells. Remaining in the capture well was the enriched population of trophoblast cells. We also performed a proof-of-concept on an imaging and picking platform to show the ability to pick single trophoblast cells for whole genome amplification. This is the first study to use cell settling for enriching trophoblast cells from a heterogeneous cervical cell population. Ultimately, we provide a technique that is quick, inexpensive, minimizes cell loss, and results in retrieval of individual trophoblast cells.

## Material and Methods

### Patient selection

Approval for enrolling patients for non-invasive prenatal sample acquisition, including endocervical swabs, was given by the Biomedical Research Alliance of New York Institutional Review Board (BRANY IRB) (File # 14-02-450-408). Written informed consent was obtained from the participating women and all personal information was removed from the specimen prior to receiving. Women in their 5^th^ to 20^th^ week of pregnancy were selected for sampling. All studies were performed in accordance with relevant guidelines and regulations.

### Endocervical sampling

Retrieval of trophoblast cells from the endometrial canal was performed using a Cytobrush and following standard Pap test protocol. Cells were rinsed from the cytology brush into 20 mL of ThinPrep^®^ PreservCyt^®^ (Hologic, Marlborough, MA) fixative solution immediately after removal from the cervix. The material was sent to the lab for experiment use within 24 h of collecting. 15 samples were used as received unless noted otherwise.

### Trophoblast cell culture

JEG-3 (ATCC, Manassas, VA), TCL-1 (kindly donated by Women & Infants Hospital Kilguss Research Institute, Providence, RI), and HTR-8 (kindly donated by Women & Infants Hospital Kilguss Research Institute, Providence, RI) trophoblast cell lines were cultured in Rosewell Park Memorial Institute medium 1640 (Gibco, Waltham, MA) (RPMI supplemented with 10% (v/v) fetal bovine serum (FBS) (Corning, Corning, NY) and 1% (v/v) penicillin streptomycin) at 37 °C under 5% CO_2_. During cell passage, cells were put in PreservCyt^®^ solution at a concentration of 100,000 cells/mL and stored at 4 °C for use up to 2 months. JEG-3, TCL-1, or HTR-8 cells were added into a cervical specimen at a known concentration to investigate optimized enrichment.

### Microscopy

#### Confocal microscopy

Confocal microscopy (Nikon Instruments A1 Confocal Laser Microscope with NIS Elements software) was used to examine geometries of cervical and JEG-3 cells. Cells in PreservCyt^®^ were dried on glass slides and rinsed 3× with 1× phosphate buffered saline (PBS). 0.1% triton X-100 in 1× PBS was incubated for 3–5 min and subsequently rinsed 3× with 1× PBS. 10% normal goat serum was incubated for 30 min at room temperature for blocking. 2.5% Alexa Fluor 532 phalloidin (Life Technologies, Carlsbad, CA) in 1% normal goat serum was incubated on the slides for 20 min at room temperature. A final wash with 1× PBS was performed and the slides were mounted with Vectashield antifade mounting media with 4′, 6-diamidino-2-phenylindole (DAPI) (Vector laboratories, Burlingame, CA). Images were processed using National Institutes of Health Image J software (version 2.0.0-rc-65/1.52b).

#### Epifluorescence microscopy

Adhered cells and the supernatant of cells were imaged using a Nikon Ti-E inverted fluorescent microscope with NIS Elements software. At least 25 images of the adhered cells were obtained to quantify an average number of adhered trophoblast and cervical cells. Trophoblast amounts less than 250 cells were counted while imaging.

### Optimization of *in vitro* trophoblast cell line enrichment from cervical sample

The cell enrichment process was carried out using a polystyrene 24 well plate (non-tissue culture treated, surface area of 1.9 cm^2^/well) as the capture well surface. Cells and controls were introduced to these capture wells and allowed to settle for differing times to determine the timescale allowing optimal separation of cervical and fetal cells. JEG-3 cells were used to optimize this process. Prior to introducing cells to the capture wells, JEG-3 cells stored in PreservCyt^®^ at a stock concentration of 100,000 cells/mL were centrifuged using a Labnet Prism microcentrifuge at 10,000 × g for 5 min. The supernatant was removed, and the cells were incubated with DAPI for 5 min. Controls containing 1,000 DAPI stained JEG-3 cells in 600 μL PreservCyt^®^ were examined for cell settling for 0, 4, 20, and 60 min. To determine the capture well incubation time allowing optimal cell separation, DAPI stained JEG-3 cells were mixed with cervical samples and incubated in the capture wells for 0, 0.5, 1, 2, 4, 8, 16, or 60 min at a constant JEG-3 density of 1,000 cells per well. The JEG-3 cells (10 μL in PreservCyt^®^) were added to either 600 μL of cervical sample or 600 μL of cervical sample diluted with 2.4 mL of PreservCyt^®^ (yielding a final volume of ~3 mL). The different volumes lead to different liquid heights; at 600 μL the well is filled to a 5 mm height, while at 3 mL the well is filled to a 20 mm height. After the capture time was completed, the supernatant was immediately removed and placed into a new well for imaging. Once the optimized separation time and height of settling were determined, JEG-3 densities of 15, 100, 250, 500, and 1,000 cells per well were studied.

The effect of washing the cells with fresh PreservCyt^®^ or 1× PBS was also examined, as it was hypothesized that these washes would remove mucus content. Cervical and JEG-3 cells were centrifuged at 2,000 rpm for 5 min, the supernatant was removed, and the cell pellet was re-suspended in fresh PreservCyt^®^. This wash procedure was performed a total of 3 times. JEG-3 cells were then incubated with DAPI prior to enrichment with the capture surface. The optimized settling time and 1,000 cells per well seeding density was used. The effect of solution density was investigated with JEG-3 cell settling at the optimized time point in Ficoll Type 400, 20% in H_2_O solution. The JEG-3 cells (10 μL in PreservCyt^®^) were added to 600 μL of Ficoll and allowed to settle.

HTR-8 and TCL-1 cells stored in PreservCyt^®^ at a stock concentration of 100,000 cells/mL were centrifuged and DAPI stained as previously performed with JEG-3 cells. The optimized time determined for JEG-3 separation from the cervical cells was used with an HTR-8 or TCL-1 density of 1,000 cells per well (10 μL in PreservCyt^®^) in 600 μL of cervical sample. After the capture time was completed, the supernatant was removed and placed into a new well for imaging.

### CyteFinder for trophoblast enrichment analysis

RareCyte CyteFinder (RareCyte, Seattle WA) was used to analyze JEG-3 capture and pick individual cells. A density of 100 JEG-3 cells per well with a cervical sample concentration of ~100,000 cells/mL was used for performing cell settling at the optimized time condition identified for JEG-3 cell separation as described above in *Optimization of in vitro trophoblast cell line enrichment from cervical sample*. 600 μL of sample was allowed to settle. Additionally, conditions with cervical cell washes and incubations were performed. The cervical sample was centrifuged at 2,000 rpm and washed 3× in fresh PreservCyt^®^ before spiking with JEG-3 cells for settling. The cervical sample and JEG-3 cells were also allowed to incubate for at least 1 h at room temperature prior to settling.

In order to perform imaging and picking on the CyteFinder, the captured cells required transfer from the 24 well polystyrene plate to a slide surface compatible with the instrument. After settling, the capture surface cells were immediately rehydrated with 600 μL of fresh PreservCyt^®^ and vigorously pipetted to remove cells from the bottom of the polystyrene well plate. Cells were transferred to a Shandon™ coated cytoslide (ThermoFisher Scientific, Waltham, MA) and dried at 50 °C. Purified mouse anti-human HLA-G denatured (BD Biosciences, San Jose, CA) at 1:100 in antibody diluent (Dako, Agilent Technologies, Inc., Santa Clara, CA) was incubated on the slides for 30 min at room temperature. The slides were rinsed with 1× PBS and Alexa Fluor 488-conjugated AffiniPure Goat Anti-Mouse IgG (H + L) (Jackson ImmunoResearch, West Grove, PA) at 1:250 in antibody diluent was incubated for 15 min at room temperature. The slides were then rinsed with 1× PBS and DAPI was incubated for 5 min. A final rinse was performed with 1× PBS and then the slides were mounted with CyteMount Mounting Media (RareCyte, Seattle, WA).

#### CytePicker for individual trophoblast cell picking

RareCyte CytePicker (RareCyte, Seattle, WA) was used to pick JEG-3 and potential real trophoblasts found during the CyteFinder process. Coverslips were soaked off of the slides in warmed PBS at 37 °C. A CytePicker needle was used to remove the trophoblast cells of interest. The cell was then removed from the slide surface and dispensed into 5 μL of PCR grade water.

### Whole genome amplification and gender polymerase chain reaction

Whole genome amplification (WGA) (SMARTer PicoPLEX WGA kit, Takara Bio Inc., Kusatsu, Shiga Prefecture, Japan) was performed by adding 5 μL of freshly-prepared extraction cocktail to the 5 μL single cell picked sample. The sample was incubated in a thermal cycler at 75 °C for 10 min, then 95 °C for 4 min, and 12 °C on hold. The pre-amplification cocktail components were then combined and mixed well with 24 μL of pre-amplification buffer and 1 μL of pre-amplification enzyme. 5 μL of this cocktail was added to each cell and incubated in a thermal cycler with one 2 min cycle at 95 °C, 12 cycles with 15 s at 95 °C, 50 s at 15 °C, 50 s at 25 °C, 30 s at 35 °C, 40 s at 65 °C, and 40 s at 75 °C. A final cycle at 4 °C was put on hold. The amplification cocktail was then mixed. 60 μL of the amplification cocktail was added to 15 μL of pre-amplification product and mixed. The sample was then amplified with one 2 min cycle at 95 °C, and 14–16 cycles with 15 s at 95 °C, 1 min at 65 °C, and 1 min at 75 °C. The WGA product was stored at −20 °C.

Gender quantitative real-time polymerase chain reaction (PCR) was performed with TaqMan Fast Universal PCR Mix (ABI-4401631), Vic labeled ribonuclease P (RNAseP) 20X Control Primers (ABI-4401631), FAM labeled *SRY* 20X Primer Mix (ABI-4400291), and *DYS14* primers and probe with F: GAGCAGGCGTGGGTACTATTG, R: GTCTGCTGCTCGGCATCAC, P: /ROX/CCTGCATGCGGCAGAGAAACCC/IBRQ/. The *DYS14* primer mix was made with 2 μM of each primer and probe. The PCR mix was made with 10 μL of *SRY* primers, 10 μL RNAse primers, 5 μL *DYS14* primers and 65 μL deionized water. BioRad CFX96 real time PCR was run with cycling at 95 °C for 5 min, 95 °C for 15 s, 60 °C for 1 min. 50 cycles were performed for each run.

### Statistical analysis

All studies were performed in triplicate at minimum. Results are shown as average ± standard deviation. All results are from at least n = 3. Where applicable, a two-tailed t-test or two-way analysis of variance (ANOVA) with Tukey’s post-hoc analysis and 95% confidence interval was performed where *p < 0.05, **p < 0.01, ***p < 0.001, and ****p < 0.0001.

## Results and Discussion

### JEG-3 and cervical cell geometry

**Figure 2:**
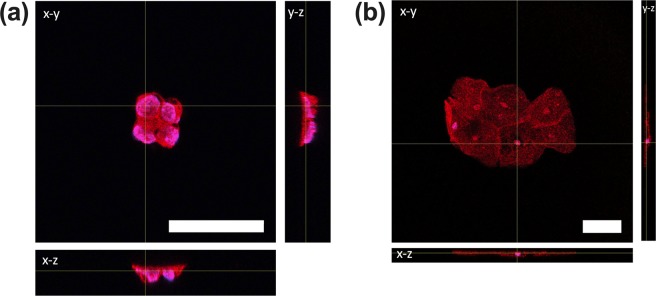
Confocal images of (**a**) JEG-3 and (**b**) cervical cells. Cells were stained with phalloidin (red) and DAPI (blue). Orthogonal views are shown with X-Y, Y-Z, and X-Z planes. The lines represent the view point. Scale bar = 50 μm.

In this work, we developed a label-free technique for trophoblast cell enrichment. Prior to investigating cell separation, we examined physical differences with cervical and trophoblast cells. To characterize the geometry of each cell type, the actin cytoskeleton and nuclei of JEG-3 and cervical cells were stained with phalloidin and DAPI, respectively. We observed that JEG-3 cells (Fig. 2a) exhibited a spherical shape while individual cervical cells and cervical cell aggregates (Fig. 2b) exhibited a more pancake-like morphology. The JEG-3 cells were significantly smaller (dimensions of 13 ± 1 × 11 ± 1 × 8 ± 1.5 μm) than single cervical cells (dimension of 66 ± 2 × 58 ± 1.5 × 6 ± 1 μm).

The size of the JEG-3 cell nuclei relative to the cell body was considerably larger than the cervical cells. The diameter of the JEG-3 cell nucleus was found to be 10.8 ± 1 μm while the diameter of the cervical cell nucleus was 8.7 ± 1.5 μm. The percentage of the cell nuclei was then taken relative to the X dimension of the cell body, resulting in approximately 82% for JEG-3 and 13% for the cervical cells. Previous investigations demonstrate cell type affects settling velocity^20–22^. Based on the cell differences observed we hypothesize that the physical properties of cervical and JEG-3 cells may influence their gravitational settling in solution.

### Optimization of JEG-3 cell enrichment using cell settling

Cervical samples can have a variable number of trophoblast cells, resulting in patient to patient variability. To optimize workflow parameters, cervical samples were first spiked with JEG-3 cells. By doing this, the JEG-3 cells experienced the mucus content present in the cervical sample from women during pregnancy.

#### Effect of time in capture well on cell settling

**Figure 3:**
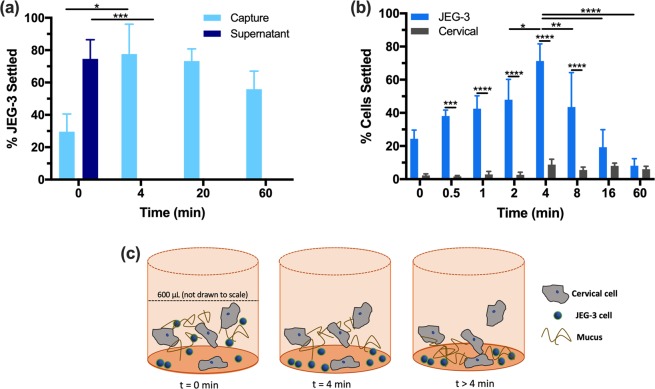
JEG-3 cell settling. (**a**) 1,000 JEG-3 cells settling in PreservCyt^®^ for 0, 4, 20, and 60 min. (**b**) 1,000 JEG-3 cells in 600 μL cervical sample for 0, 0.5, 1, 2, 4, 8, 16, and 60 min. (**c**) Schematic of the hypothesized settling of JEG-3 cells, cervical cells, and mucus over time. Two-way ANOVA with Tukey post-hoc analysis and a 95% confidence interval was performed. n ≥ 3; *p < 0.05; **p < 0.01; ***p < 0.001; ****p < 0.0001.

Our workflow, described in Fig. 1 Step 3, requires a set amount of time in a capture well before the supernatant is removed. First, a control study was performed with 1,000 JEG-3 cells settling in only PreservCyt^®^ (Fig. 3a). We observed that on average 80% of JEG-3 cells settled in 4 min with an average velocity of 1.25 mm/min. As the cell suspension was very dilute, we assumed no hydrodynamic interactions between cells. The observed 10–20% unsettled cell population can be attributed to cell adsorption onto the walls of the wells utilized during capture. It appears that the cell interaction with the polystyrene surface plays a significant role in the JEG-3 surface capture. After 4 min there was no significant change in JEG-3 settling at longer time points. Next, cell settling was performed with 1,000 JEG-3 cells in 600 μL of cervical sample. To determine the optimal settling time, cervical samples with 1,000 JEG-3 cells were incubated for 0, 0.5, 1, 2, 4, 8, 16, and 60 min in the capture well before removing the supernatant (Fig. 3b). At 4 min we observed the largest number of JEG-3 cells remaining on the surface, an average of 713 ± 103 cells from the original 1,000 cells incubated.

We also observed after 4 min, the number of JEG-3 cells captured at the well surface was significantly decreased at 16 and 60 min. At these time points no significant difference was observed between the percentage of cervical cells and the percentage of JEG-3 cells adhered to the capture surface. Our control experiment with JEG-3 cells showed a plateau after 4 min of cell settling, so the observed decrease was due to partial recovery of JEG-3 cells in the presence of the cervical sample content. We hypothesize the decrease in JEG-3 capture following 4 min is due to the highly viscous and “sticky” nature of the mucus in the cervical sample, which itself continues to settle over time, as depicted in Fig. 3c. Once a critical amount of mucus accumulates on the surface of the capture well, it obscures further settling or detection of cells on the surface. As the JEG-3 cells and cervical squamous cells exhibited different geometries, their settling behavior is expected to differ. The ability for JEG-3 cells to settle before the cervical cells may be due to a combination of Stoke-dependent settling^22,23^, capture well polystyrene surface, and the trophoblast cell invasive nature^24–27^.

Overall, we observed an optimized separation time of 4 min. The workflow was able to remove an average of 91 ± 3% of the cervical squamous cells from the sample, while capturing an average of 71 ± 10% of the JEG-3 cells. For this time point, the initial JEG-3 cell population was 1,000 cells while the initial cervical cell population was ~72,000 cells. After settling, 713 ± 103 JEG-3 cells were recovered with 6,320 ± 2,390 cervical cells, resulting in an unprecedented JEG-3 enrichment of 707 ± 330%.

#### Effect of volume on cell settling

**Figure 4:**
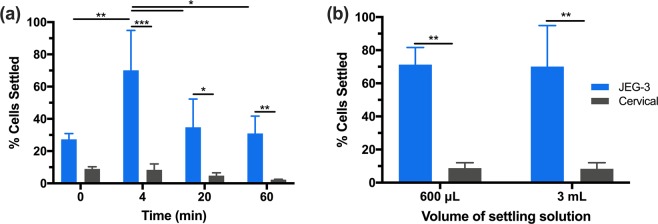
(**a**) JEG-3 and cervical cell settling over time with a total volume of 3 mL and height of 20 mm in the well. (**b**) 4 min time of settling for 600 μL and 3 mL of settling volume. Two-way ANOVA with Tukey post-hoc analysis and a 95% confidence interval was performed. n ≥ 3; *p < 0.05; **p < 0.01; ***p < 0.001; ****p < 0.0001.

Having established JEG-3 separation behavior at a volume of 600 μL (~5 mm height), we next studied how JEG-3 settling changes with an increased volume of 3 mL (~20 mm height). We performed settling of 1,000 JEG-3 cells in cervical samples for 0, 4, 20, and 60 min (Fig. 4a). Our results again indicated an optimal settling time of 4 min with 70 ± 25% JEG-3 settling and 8.4 ± 3.6% cervical cells remaining on the capture surface. These results are similar to the optimal capture observed with 600 μL, where the volume of settling did not result in significant differences in JEG-3 capture at 4 min (Fig. 4b). Once again, settled mucus concentrates after 4 min, which disallowed or obscured further settling or detection of cells. With the 3 mL volume we still observed a trend of decreased JEG-3 cell settling after 4 min, but less pronounced compared to the 600 μL volume. With the 3 mL volume, 20 and 60 min settling resulted in a significant difference between the JEG-3 and cervical cell settling, which was not observed for the 600 μL volume. Therefore, if it is necessary to extend the time for optimal settling increasing the volume was shown to help. For the current study, because there was no significant difference in JEG-3 capture and cervical cell elimination at 4 min, we continued our optimization with 600 μL.

#### Effect of cell number on cell settling

**Figure 5:**
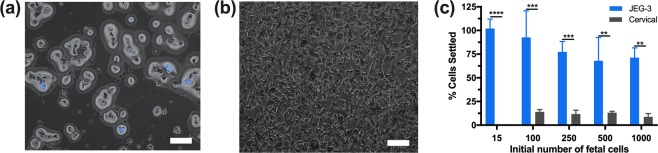
(**a**) Capture well JEG-3 cells are DAPI stained and can be easily distinguished from cervical cells. (**b**) Population of cervical squamous cells removed with the supernatant. Scale bar = 200 μm. (**c**) Effect of number of JEG-3 cells with cervical sample settling for 4 min. Two-way ANOVA with Tukey post-hoc analysis and a 95% confidence interval was performed. n ≥ 3; *p < 0.05; **p < 0.01; ***p < 0.001; ****p < 0.0001.

Next, it was important to test if there was a dependence on the initial number of JEG-3 cells in the cervical sample. We studied 15, 100, 250, 500, and 1,000 JEG-3 cells in a cervical sample with 4 min settling. Figure 5a,b show a representative image for the study performed with 1,000 JEG-3 cells per well. The JEG-3 cells remaining on the capture surface (Fig. 5a) are observed with the blue DAPI stain. While cervical cells still remain on the capture surface, a large number of cervical cells are removed in the supernatant (Fig. 5b). Figure 5c shows no significant difference in the percentage of JEG-3 cells settled to the capture surface at the tested cell numbers.

#### Effect of washing cells on cell settling

Previous studies have first removed mucus from cervical samples by PBS washing before performing isolation techniques^6^. Next, we washed the cervical sample with fresh PreservCyt^®^ or 1× PBS before cell settling to determine the role of mucus (as depicted in Fig. 1 Step 2).

**Figure 6:**
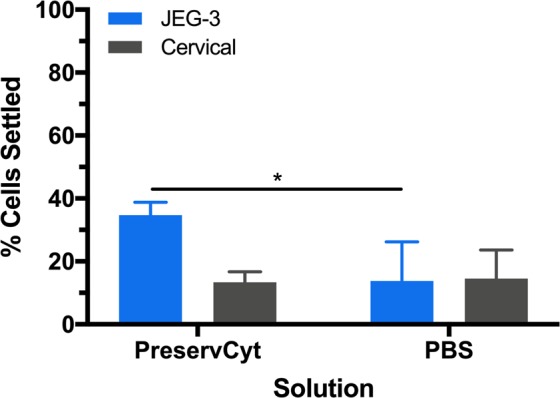
Cervical sample rinsed with PreservCyt^®^ or PBS before 4 min cell capture study. Two-way ANOVA with Tukey post-hoc analysis and a 95% confidence interval was performed. n ≥ 3; *p < 0.05; **p < 0.01; ***p < 0.001; ****p < 0.0001.

When washed with PreservCyt^®^, only 34 ± 4% of JEG-3 cells settled (Fig. 6) compared to the 71 ± 10% of JEG-3 settled when not washed (Fig. 3b). With the PreservCyt^®^ wash no significance was observed between JEG-3 and cervical cell settling. The percentage of cervical cells on the capture surface was not significant between the washed (13 ± 3%, Fig. 6) and unwashed (9 ± 3%, Fig. 3b) conditions. When washed with PBS, the difference in the percentage of settling for JEG-3 cells and cervical cells was not statistically significant. The 13 ± 12% of JEG-3 settling after PBS washing was significantly lower than the 34 ± 4% of JEG-3 settling after PreservCyt^®^ washing.

These results point to the presence of mucus having a significant effect during the enrichment workflow. Previous work has indicated that cervical mucus impacts the diffusion of macromolecules and particles^28,29^. These studies have focused on delivery across the cervical mucus barrier. It was noted that while some particles, such as the human papilloma virus and globular proteins diffuse as rapidly in the mucus as in saline, other particles such as the herpes simplex virus can colocalize with the mucus^28^. Differences in surface modification of particles, such as polymer molecular weight, has also been shown to influence their interaction with cervical mucus^29^. While the cervical and JEG-3 cells are significantly larger than these previously studied particles, differences in the cell structure (e.g. geometry, surface proteins) can influence cervical mucus interactions. Based on our results we suggest that the mucus content either aids in the JEG-3 cell settling and/or restricts cervical cell settling. For our workflow, it is optimal to use the sample as received and not perform any washes or solution change in order to obtain maximal JEG-3 capture and enrichment.

#### Effect of solution density on cell settling

**Figure 7:**
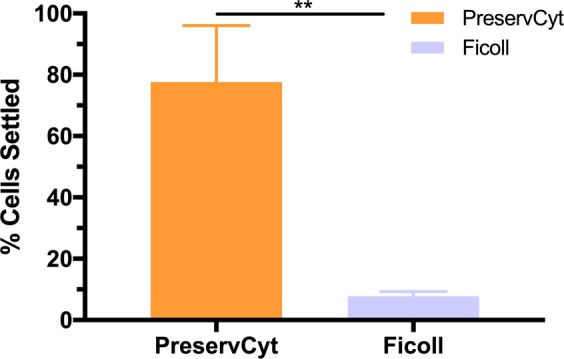
Percentage of JEG-3 cells settled after 4 min in PreservCyt^®^ and Ficoll. Two-tailed t-test with 95% confidence interval was performed. n ≥ 3; *p < 0.05.

JEG-3 settling was then studied for 4 min in a Ficoll solution. Ficoll has a density of 1.055–1.075 g/mL compared to methanol with a density of 0.791 g/mL, which comprises 30–60% of PreservCyt^®^. Compared to the JEG-3 control with 4 min of settling in PreservCyt^®^, which captures 78 ± 18% JEG-3 cells, 4 min of settling in Ficoll resulted in 7.8 ± 1.5% of JEG-3 cell capture (Fig. 7). Thus, the solution density of settling plays a role in the percentage of JEG-3 capture on the surface.

Ficoll is a highly branched polymer formed by copolymerization of sucrose and pichlorohydrin^30^. It is possible Ficoll may act similarly to mucus content in solution as both Ficoll and mucin are gel-like substances^30,31^. An amount of Ficoll is adsorbed to the capture surface from the start of JEG-3 settling. This is dissimilar to the cervical sample where mucus is suspended in the PreservCyt^®^ solution. Due to the concentrated Ficoll solution, we expected JEG-3 capture with Ficoll to be similar to JEG-3 capture after mucus settling. From our previous experiments, this occurs after 8 min (Fig. 3b). The resulting JEG-3 cell capture in Ficoll is similar to the 8.2 ± 4.2% JEG-3 settling at 60 min in the cervical sample (Fig. 3b), when mucus content at the capture surface is greatest. This further suggests presence of gel-like substances (Ficoll or mucus) at the capture surface restricts JEG-3 retrieval.

#### Effect of trophoblast cell line on cell settling

**Figure 8:**
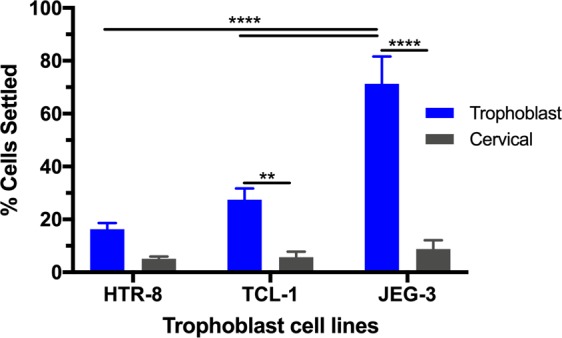
Comparison of trophoblast cell lines settling for 4 min. Two-way ANOVA with Tukey post-hoc analysis and a 95% confidence interval was performed. n ≥ 3; *p < 0.05; **p < 0.01; ***p < 0.001; ****p < 0.0001.

Using 4 min capture time, HTR-8 and TCL-1 trophoblast cell lines were used to investigate cell separation dependence on cell line (Fig. 8). HTR-8 and TCL-1 trophoblast cell lines are representative of first and third trimester trophoblasts, respectively^32,33^. Compared to the 71 ± 10% settling of the JEG-3 cells to the capture surface, the HTR-8 and TCL-1 resulted in 16 ± 2% and 27 ± 4%, respectively. The JEG-3 capture was significantly different from the HTR-8 and TCL-1 cells. As was observed with the JEG-3 cells, a significant number of cervical cells was still removed from the population with the HTR-8 and TCL-1 spiked samples. It is possible that the ideal 4 min capture time may change for these additional cell lines.

JEG-3 cells have commonly been used as a model EVT cell line^34^. Previous investigations have studied the villous versus extravillous nature of HTR-8 and TCL-1 cells. HTR-8 cells originated from first trimester villous explants^33^ while TCL-1 cells were established from term placenta^32^. Investigations have looked at the EVT signature of these cell lines, and have found TCL-1 cells to have stronger EVT expression compared to HTR-8 cells^35^. This previous literature and our results suggest that the degree of EVT features on the cells may influence their settling in solution.

### Enrichment of JEG-3 and trophoblasts for single cell picking and whole genome amplification

**Figure 9:**
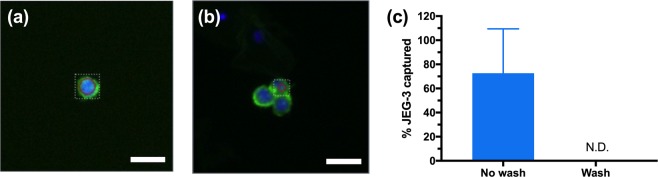
CyteFinder. (**a**) Representative JEG-3 cell. (**b**) Representative JEG-3 cells. (**c**) Percentage of JEG-3 captured with and without PreservCyt^®^ wash. The wash condition had no detectable (N.D.) cells found by the CyteFinder instrument. n = 3; Scale bar = 20 μm.

A hundred JEG-3 cells were spiked into a cervical sample with a concentration of ~100,000 cells/mL in order to more closely represent trophoblast concentration in cervical samples. First, we investigated settling of the JEG-3 cells at 4 min. After settling, the captured cells were transferred onto a coated slide. HLA-G staining was performed and analyzed by the CyteFinder (Fig. 9a,b). Images from the CyteFinder showed HLA-G labelled JEG-3 cells with single JEG-3 cells and cell clumps observed. Cervical cells that lack HLA-G are also observed in Fig. 9b as DAPI stained nuclei only. We observed a JEG-3 capture of 73 ± 36% (Fig. 9c), which is comparable to our previous optimization results. The role of incubating the JEG-3 and cervical cells together for a maximum of 1 h was also investigated for detection of captured JEG-3 cells using the CyteFinder (Supplementary Fig. S1).

The cervical sample was then washed 3 times with fresh PreservCyt^®^ before settling with JEG-3 cells. We expected that these rinses would result in a reduced amount of cervical mucus present in the sample, similar to results from section *Effect of washing cells on cell settling*. After the wash, the CyteFinder instrument could not detect (N.D.) enough cells on the slide surface to produce a report. This result corresponds with our previous results and discussion on the role of mucus.

#### Cell picking

**Figure 10:**
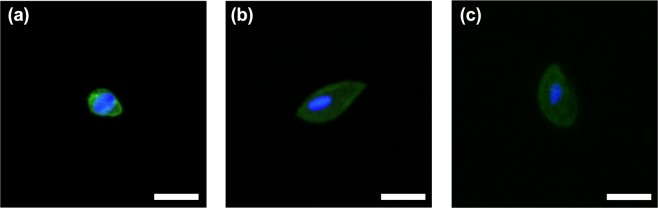
Three cells of interest isolated using CytePicker (**a**) JEG-3 cell control. (**b**) Potential real trophoblast. (**c**) Potential real trophoblast. Scale bar = 20 μm.

JEG-3 and potential real trophoblast cells of interest found during the CyteFinder were then picked from the slide surface (as depicted in Fig. 1 Step 4). A control JEG-3 cell (Fig. 10a) and two trophoblast cells (Fig. 10b,c) were chosen for this proof-of-concept. Observed in each of these images was the ability to pick only these single cells with no cervical contamination in the surrounding area. Figures presented here were magnified using Image J software. Original images from the CyteFinder are presented in Supplementary Fig. S2, which further demonstrate the boundary area free of cervical cells.

#### Whole genome amplification and gender polymerase chain reaction

For proof-of-concept testing, WGA with PicoPLEX was performed for a trophoblast cell and a JEG-3 cell picked using the CyteFinder (as depicted in Fig. 1 Step 4). Following genome amplification, gender PCR was performed. RNAse P signal demonstrated DNA presence in both the trophoblast and JEG-3 cell (Table 1). In this particular test case, the *DYS14* and *SRY* Y chromosome markers, were not present for the putative native trophoblast cell as observed for the JEG-3 control. Although this test cannot confirm the picked trophoblast as fetal in origin due to the lack of Y chromosome markers as this suggests a female fetus, this proof-of-concept shows the ability of our enrichment method to be applied to obtaining the genetic material from a single cell obtained from the cervical sample. Further, the strong Y chromosome signal observed for the JEG-3 cell demonstrates that cervical content is not masking the detection.

**Table 1:** Trophoblast and JEG-3 gender PCR.

Cell	Cycle Threshold		
	RNAse P	*DYS14*	*SRY*
Trophoblast	30	Fail	Fail
JEG-3	20.54	16.13	21.26

## Conclusions

In this study we provide an optimized workflow for enriching trophoblast cells from a heterogeneous cervical cell population. Our enrichment method is inexpensive and label-free. Using JEG-3 cells, we conclude that enrichment is possible with removing at least 90% of squamous cervical cells while capturing at least 70% of JEG-3 cells at an optimal 4 min settling time. This results in a 707 ± 330% enrichment of trophoblast cells from the heterogenous population. This method is not dependent on initial trophoblast cell number and this process can be further scaled to larger volumes. We found that using the cervical sample as collected and sent to the lab was the most advantageous for enriching JEG-3 cells using our method, which makes this a streamlined process. As the cervical cell settling was observed to be similar in different solutions tested, we hypothesize that the polystyrene surface has a significant role restricting the cervical cell settling. However, as JEG-3 cell settling changes with PreservCyt^®^ and PBS washes and with Ficoll, we hypothesize the mucus content in the PreservCyt^®^ solution is significant for trophoblast capture.

These results were further confirmed with our proof-of-concept studies using CyteFinder and CytePicker. We isolated single JEG-3 and native trophoblast cells found during the CyteFinder analysis and analyzed the DNA content through WGA and PCR. Ultimately, these results show that our workflow can make new isolation techniques, such as cell picking, more efficient and effective.

## Supplementary information

Supplementary Information

## Data Availability

Data generated during the current study are available from the corresponding author on reasonable request.
