# Supplementary material for: A Rapid Method for Label-Free Enrichment of Rare Trophoblast Cells from Cervical Samples
Source: Sci Rep. 2019 Aug 20;9:12115. doi: 10.1038/s41598-019-48346-3 (PMC6702343; doi:10.1038/s41598-019-48346-3)
Supplement: Supplementary file 1 — Supplementary Information [file 41598_2019_48346_MOESM1_ESM.docx]

**Supplementary Information**

**A rapid method for label-free enrichment of rare trophoblast cells from cervical samples**

Christina M. Bailey-Hytholt^1^, Sumaiya Sayeed^1^, Morey Kraus^2^, Richard Joseph^2^, Anita Shukla^1^ and Anubhav Tripathi^1*^

*^1^Center for Biomedical Engineering, School of Engineering, Brown University, Providence, RI 02912*

*^2^PerkinElmer, 940 Winter St, Waltham, Massachusetts 02451.*


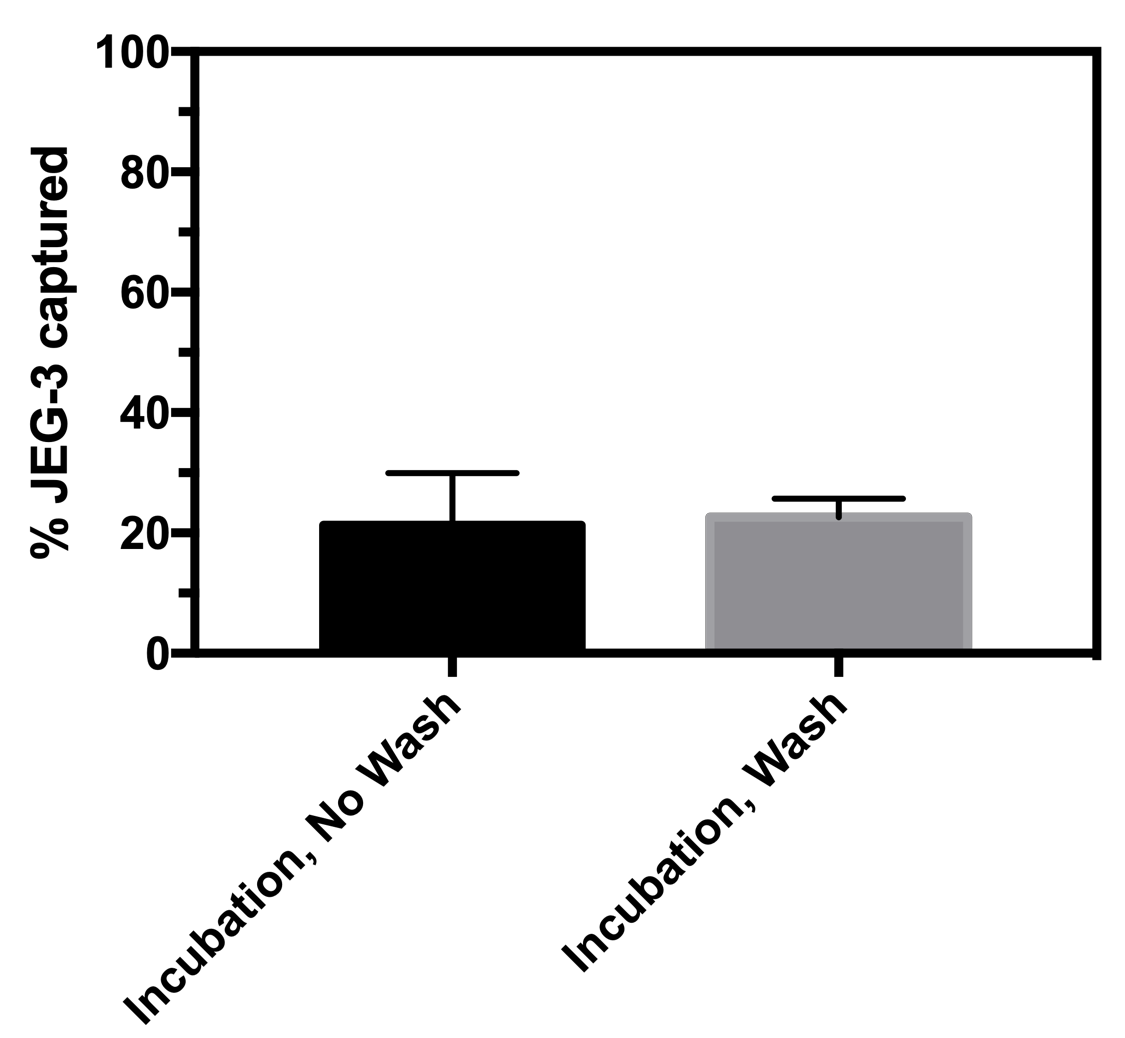


**Supplementary Figure S1:** JEG-3 and cervical sample were allowed to incubate together for up to 1 h maximum time prior to performing the separation. This resulted in reduced percentage of JEG-3 recovery from the initial spiked in JEG-3 cells. JEG-3 cells were also allowed to incubate with the cervical sample for a period of time prior to settling after the cervical sample was washed 3x with fresh PreservCyt^®^.


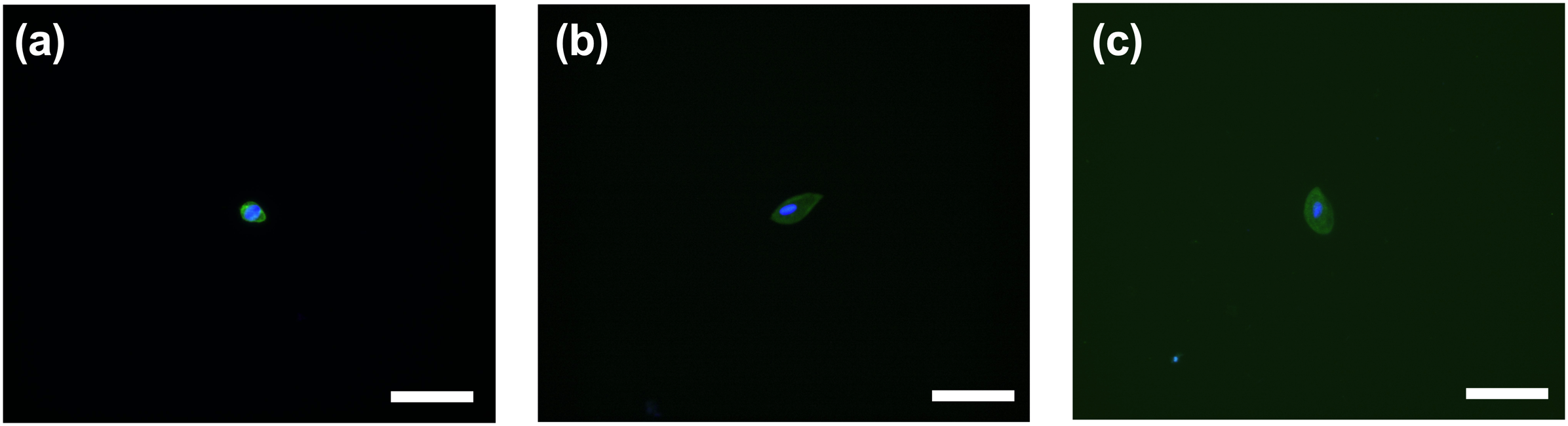


**Supplementary Figure S2:** Images from the CyteFinder with no Image J alterations. Three cells of interest that were picked using CyteFinder from the slide surface as a proof-of-concept trophoblast isolation. All cells were from the no incubation and no wash condition **(a)** JEG-3 cell control. **(b)** Potential real trophoblast. **(c)** Potential real trophoblast. Scale bar = 50 μm.
